# Microcolony Imaging of *Aspergillus fumigatus* Treated with Echinocandins Reveals Both Fungistatic and Fungicidal Activities

**DOI:** 10.1371/journal.pone.0035478

**Published:** 2012-04-19

**Authors:** Colin J. Ingham, Peter M. Schneeberger

**Affiliations:** 1 Department of Medical Microbiology and Infection Control, Jeroen Bosch Hospital, ‘s-Hertogenbosch, The Netherlands; 2 Laboratory of Microbiology, Wageningen University, Wageningen, The Netherlands; 3 MicroDish BV, Wageningen, The Netherlands; Universidade de Sao Paulo, Brazil

## Abstract

**Background:**

The echinocandins are lipopeptides that can be employed as antifungal drugs that inhibit the synthesis of 1,3-β-glucans within the fungal cell wall. Anidulafungin and caspofungin are echinocandins used in the treatment of *Candida* infections and have activity against other fungi including *Aspergillus fumigatus*. The echinocandins are generally considered fungistatic against *Aspergillus* species.

**Methods:**

Culture of *A. fumigatus* from conidia to microcolonies on a support of porous aluminium oxide (PAO), combined with fluorescence microscopy and scanning electron microscopy, was used to investigate the effects of anidulafungin and caspofungin. The PAO was an effective matrix for conidial germination and microcolony growth. Additionally, PAO supports could be moved between agar plates containing different concentrations of echinocandins to change dosage and to investigate the recovery of fungal microcolonies from these drugs. Culture on PAO combined with microscopy and image analysis permits quantitative studies on microcolony growth with the flexibility of adding or removing antifungal agents, dyes, fixatives or osmotic stresses during growth with minimal disturbance of fungal microcolonies.

**Significance:**

Anidulafungin and caspofungin reduced but did not halt growth at the microcony level; additionally both drugs killed individual cells, particularly at concentrations around the MIC. Intact but not lysed cells showed rapid recovery when the drugs were removed. The classification of these drugs as either fungistatic or fungicidal is simplistic. Microcolony analysis on PAO appears to be a valuable tool to investigate the action of antifungal agents.

## Introduction

The echinocandins are an important class of lipopeptide antifungal compounds consisting of cyclic hexapeptides linked to a long chain fatty acid. The echinocandins inhibit cell wall synthesis, by non-competitive inhibition of the 1,3-β-glucan synthase. This is thought to occur via interaction with the Fks1 subunit of this enzyme [1]. Echinocandins are commonly used to treat infections by many *Candida* species, against which they are fungicidal. Unlike an earlier class of 1,3-beta-glucan synthase inhibitors, the liposaccharide papulocandins, the activity of echinocandins extends beyond *Candida* spp., to include *Aspergillus* spp. and *Pneumocystis carinii* [2].

*Aspergillus fumigatus* is a widespread filamentous fungus, which is both highly allergenic and an opportunistic pathogen. Systemic infections by *A. fumigatus*, particularly in immunocompromised individuals, present a significant risk of mortality. Treatment is commonly with amphotericin B or triazole drugs such as itraconazole or voriconazole. Triazole resistant strains are known [3]. Caspofungin has been reported to be effective in salvage therapy for patients refractory to standard antifungal agents for invasive aspergillosis [4].

Despite 1,3-β-D-glucan being the dominant form of glucan in the cell wall of *Aspergillus* spp., echinocandins are generally considered fungistatic against these moulds rather than fungicidal [5], [6]. Chitin synthesis appears to be able to reinforce cell walls and compensate, in some instances, for echinocandin-mediated damage [2]. Caspofungin and micafungin have been suggested to have a degree of fungicidal activity based on liquid culture. This has been investigated using 5,(6)-carboxyfluorescein as a fluorogenic indicator of esterase activity in live cells and bis-(1,3-dibutylbarbituric acid)trimethine oxonol to identify dead cells [7], [8]. These studies suggest that the classification of effects of caspofungin on growth and viability in *A. fumigatus* may not be entirely straightforward. Work on this subject to date has generally been qualitative or semi-quantitative and has not looked at anidulafungin, an important drug, as a fungicidal agent.

Acquired resistance to echinocandins in pathogenic yeasts and moulds is mediated via mutations within the Fks1 subunit of the 1,3-β-D-glucan synthase [2], [5], [6], [7], [8]. Determining precise MIC values from susceptibility testing is therefore necessary for some clinical isolates, but when inhibition of growth is not necessarily complete, deriving exact MIC values can be problematic. An alternative, minimal effective concentration (MEC) based around echinocandin-induced morphology changes, has been proposed but determination of this value is rather subjective.

Culture on a porous aluminium oxide (PAO) support offers advantages in studying the effects of drugs on microorganisms over direct growth on agar or liquid culture. This ceramic material permits effective imaging of large numbers of microcolonies cultured on its upper surface – with imaging by fluorescence and scanning electron microscopy. Strips of PAO are highly porous (pore size 20 to 200 nm, 40% porosity) but only 60 µm thick. Therefore, it is possible to add or remove compounds (such as drugs or fluorogenic dyes or fixatives) rapidly but with minimal disturbance by moving the material between agars of different compositions [9]. This method has been previously used to osmotically stress bacteria and to dose microcolonies of bacteria and *Candida* spp. during rapid drug susceptibility testing [10], [11]. Microcolony-based methods combined with fluorogenic dyes and imaging may offer particular advantages with respect to studying filamentous fungi, a group of organisms for which dispersed growth in liquid culture is not necessarily the most relevant [7]. In this study, PAO culture was used to explore the lethality of caspofungin and anidulafungin to *A. fumigatus*, and also the recovery from these drugs, by imaging individual microcolonies and by quantifying the effects.

## Results

### Germination and growth of *A. fumigatus* and *A. terreus* on PAO

#### Dispersal and germination of conidia on PAO

Conidia purified from clinical isolates and a reference strains (ATCC204305) of *A. fumigatus* and *A. terreus* (Tables 1 and 2) were inoculated onto 36×8 mm strips of PAO placed on Sabouraud agar to a density of from 5 to 50 cfu/mm^2^. After incubation for 4 to 10 h at 37°C the fungi on PAO strips were stained with Fun-1/calcofluor white and the percentage germination assessed. Both germinated and ungerminated conidia of both species could be imaged and distinguished upon PAO by this method. The distribution of conidia immediately after inoculation was predominantly (>93%) of single spores. The median swelling and outgrowth times for all strains tested (Table 1) were similar on PAO placed on Sabouraud agar compared to growth directly on the same agar. This suggests conidia can be inoculated to single cfu on PAO and germination occurs with the same efficiency to that seen on agar.

**Table 1:** Comparison of germination and growth of strains of *Aspergillus* spp. studied on Sabouraud agar and on PAO placed on Sabouraud plates.

		Conidial Swelling^a^		Outgrowth^a^		Hyphal Extension (µm/h)^b^	
Strain		Agar	PAO	Agar	PAO	Agar	PAO
JBZ11	*A. fumigatus*	4.5 h	4 h	6.5 h	7 h	24.9+/−4	19.1+/−4
JBZ17	*A. fumigatus*	4 h	5 h	6 h	6 h	18.0+/−2	16.1+/−3
JBZ32	*A. fumigatus*	4.5 h	4.5 h	7 h	7 h	19.0+/−5	16.8+/−4
CWZ93	*A. fumigatus*	5 h	5 h	7 h	6.5 h	22.5+/−6	20.6+/−47
CWZ855	*A. fumigatus*	4.5 h	5 h	6.5 h	6.5 h	16+/−3	17+/−4
ATCC204305	*A. fumigatus*	4 h	4.5 h	7 h	7 h	20.0+/−4	16+/−3
CWZ59	*A. terreus*	6 h	6 h	8 h	8.5 h	11.0	8.9

a Calculated from the area of >100 conidia or microcolonies per data points; taking measurements every 30 min after inoculation. ANOVA with Tukey post hoc test was to determine first time point with a significant increase in area (P<0.05) comparing unswollen conidia at T = 0 with subsequent time points to determine swelling time. Outgrowth times were determined in a similar way, comparing the microcolony area of swollen conidia with subsequent time points .

b Mean of 20 determinations +/− S.D.

**Table 2:** Comparison of Echinocandin susceptibility of *Aspergillus* spp. studied on Sabouraud agar and on PAO placed on Sabouraud plates.

		MIC^a^ ANI		MEC^b^ ANI	MIC^a^ CASP		MEC^b^ CASP
Strain	Species	E-test	PAO		E-test	PAO	
JBZ11	*A. fumigatus*	0.06	0.06	<0.0625	0.125	0.25	0.0625
JBZ17	*A. fumigatus*	0.125	0.125	0.0625	0.25	0.25	0.0625
JBZ32	*A. fumigatus*	0.06	0.125	<0.0625	0.125	0.12	0.125
CWZ93	*A. fumigatus*	<0.008	<0.0625	<0.008	8	4	0.125
CWZ1243	*A. fumigatus*	<0.008	0.125	<0.008	>8	>32	1
ATCC204305	*A. fumigatus*	<0.008	<0.0625	<0.008	0.25	0.25	0.03
CWZ59	*A. terreus*	<0.008	0.0625	0.0008	0.5	1	0.0625

a MIC values in µg/ml.

b MEC values in µg/ml.

#### Growth of mycelia on PAO

**Figure 1:**
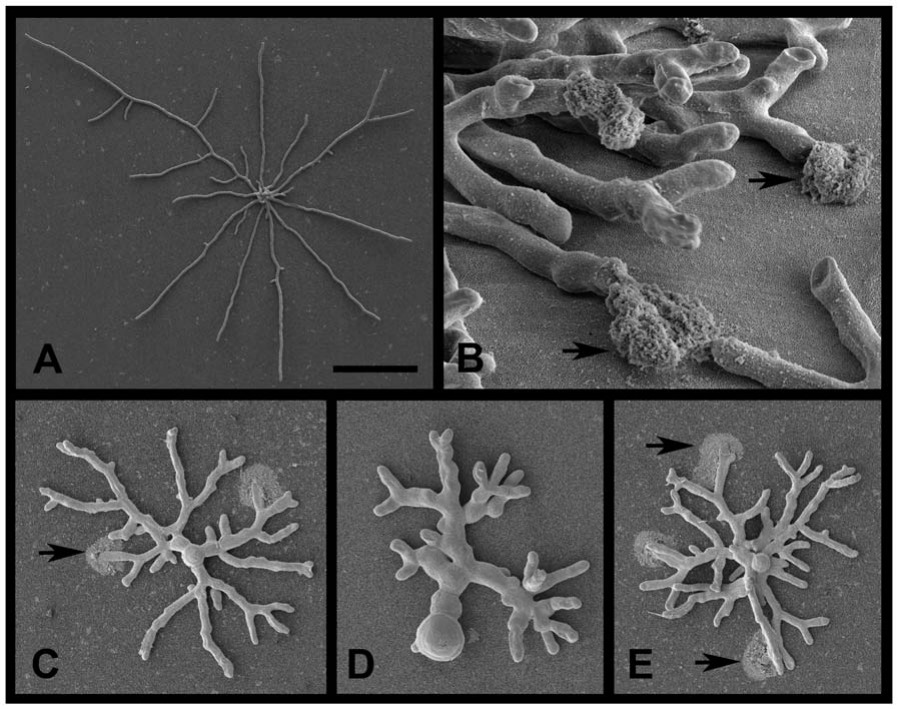
Imaging microcolonies of *A. fumigatus* JBZ11 grown on PAO with echinocandins by SEM. Culture was for 14 h at 37°C on Sabouraud medium with imaging from directly above unless stated otherwise. A: Microcolony without drugs. B: Growth with 0.0625 µg/ml anidulafungin. Imaging from the side at a 30 degree angle. C: Growth with 0.06 µg/ml anidulafungin. D: Growth with 2 µg/ml anidulafungin. E: Growth with 0.5 µg/ml caspofungin. Arrows indicate lysed hyphal tips. Scale bar in panel A indicates 90 µm. When applied to panel B, 8 µm; panel C, 30 µm; panel D, 15 µm; panel E, 30 µm.

Hyphal extension rates were calculated from transmission light microscopy, and were found to be similar on PAO compared to culture directly on the same agar medium (Table 1). Scanning electron microscopy suggested that the tips of vegetative mycelia of *A. fumigatus* were lifted off the surface of the PAO during growth (Figure 1A and 1B); observation by light microscopy supported this conclusion. Visible colonies of all strains tested were smaller on PAO compared to agar, when observed after 24 and 36 h. The formation of conidiophores on PAO was delayed by 12 to 24 h. Taken together, these data suggest that PAO is suitable for the culture of fungi to microcolonies with no nutrient limitation caused by the interposition of a 40% porous filter between agar and the fungus, the changes in surface properties of the growth surface (such as wetness or texture) having a major impact. Macroscopic growth was somewhat restricted, presumably by nutrient limitation, but this was not relevant to the microcolony studies presented in this work.

### Growth of *A. fumigatus* and *A. terreus* with anidulafungin and caspofungin

#### Effect of echinocandins on microcolony growth

**Figure 2:**
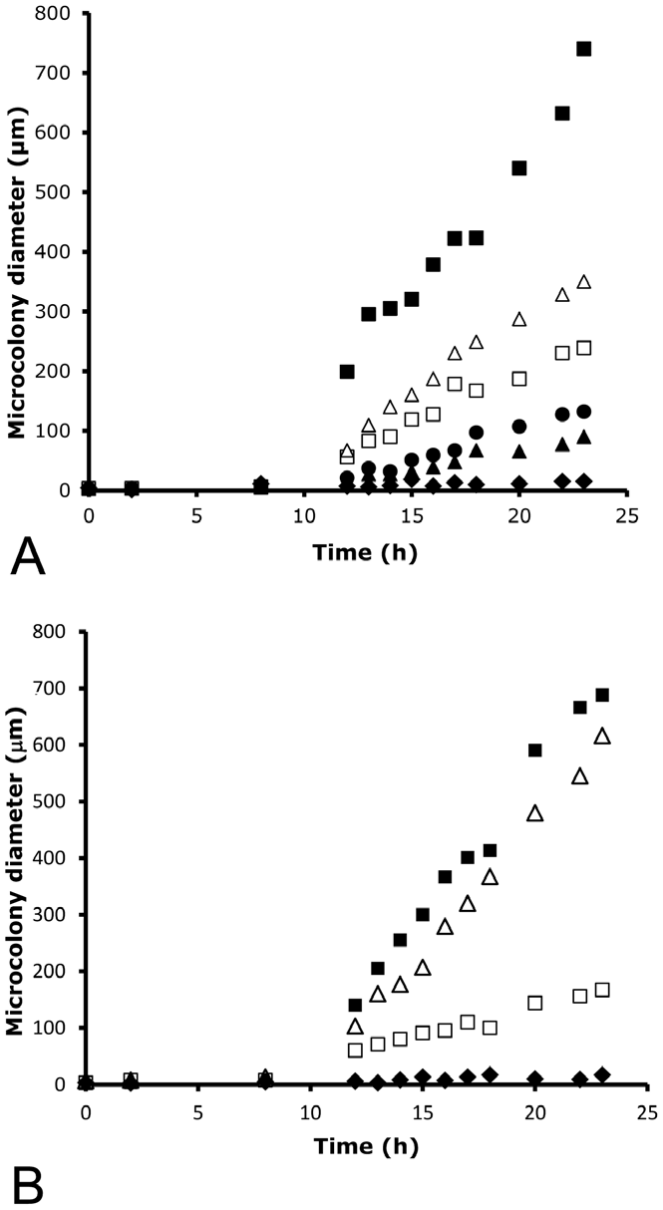
Growth of microcolonies of *A. fumigatus* grown on PAO with echinocandins. A: Microcolony growth from conidia of sensitive strain JBZ11 without drugs (solid squares), with 0.125 µg/ml anidulafungin (open squares) or 1 µg/ml anidulafungin (solid triangles), with 0.125 µg/ml caspofungin (open triangles) or 1 µg/ml caspofungin (solid circles) or 0.1 µg/ml amphotericin B (solid diamonds). B: As panel A, except using caspofungin resistant and anidulafungin sensitive strain CWZ1243.

The strains listed in Table 1 were grown from conidia for 9 h and 14 h on 0.125 to 32 µg/ml anidulafungin or caspofungin, on PAO strips. After 7 to 9 h inhibition of conidial outgrowth by both drugs could be seen but a reproducible determination of a MIC was not possible. After 14 h culture, a clear dose-dependent effect of both echinocandins on microcolony diameter was observed, allowing MIC determinations that agreed well with an established method, the E-test using 36 h culture (Table 2). Using the PAO method, even high concentrations of anidulafungin and caspofungin allowed significant, if limited, outgrowth when judged at the microcolony level (Figure 2A). In order to test if hyphal extension occurred continuously with high levels anidulafungin and caspofungin, the average microcolony diameter (using >100 microcolonies per data point) was measured at different time points over 24 h (Figure 2). These data suggested that widespread, if very slow, growth was occurring for sensitive strains in the presence of anidulafungin and caspofungin, even at a concentration >10-fold above the MIC. In contrast, amphotericin B was completely inhibitory to microcolony growth, at concentrations equal to the MIC, and above (Figure 2) Reanalysis of the same images, calculating average microcolony area instead of diameter, led to the same conclusions (data not shown). Caspofungin resistance was apparent from the higher growth rate of a resistant strain of *A. fumigatus* on PAO when dosed with this drug (Figure 2B).

#### Cell lysis by anidulafungin and caspofungin

**Figure 3:**
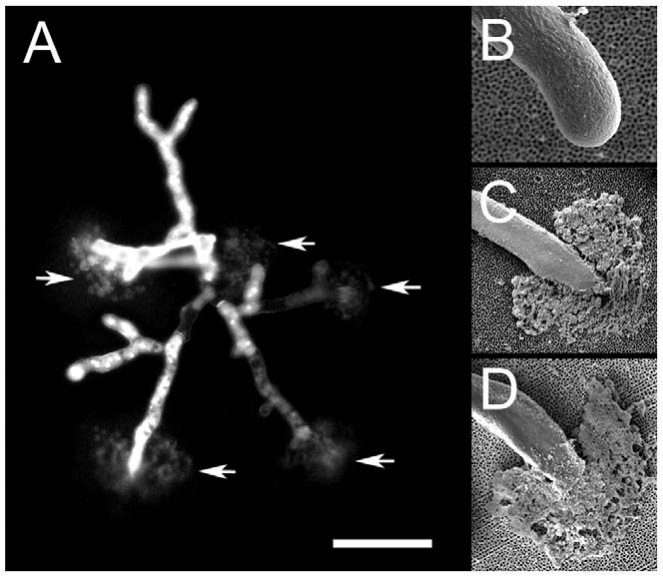
Lysis of microcolonies of *A. fumigatus* JBZ11 grown on PAO with echinocandins. Culture was for 14 h at 37°C. A: Microcolony cultured on 0.125 µg/ml anidulafungin. Staining was with Fun-1/calcofluor white and imaging by fluorescence microscopy. B: Imaging by SEM with vapour fixation of a hyphal tip grown without drugs. C: As panel B except growth with 0.125 µg/ml anidulafungin. D: As panel C, except growth with 0.125 µg/ml caspofungin. Arrows indicate lysed hyphal tips. Scale bar in panel A indicates 20 µm. When applied to panel B, 3 µm; panel C, 4 µm; panel D, 4 µm.

**Figure 4:**
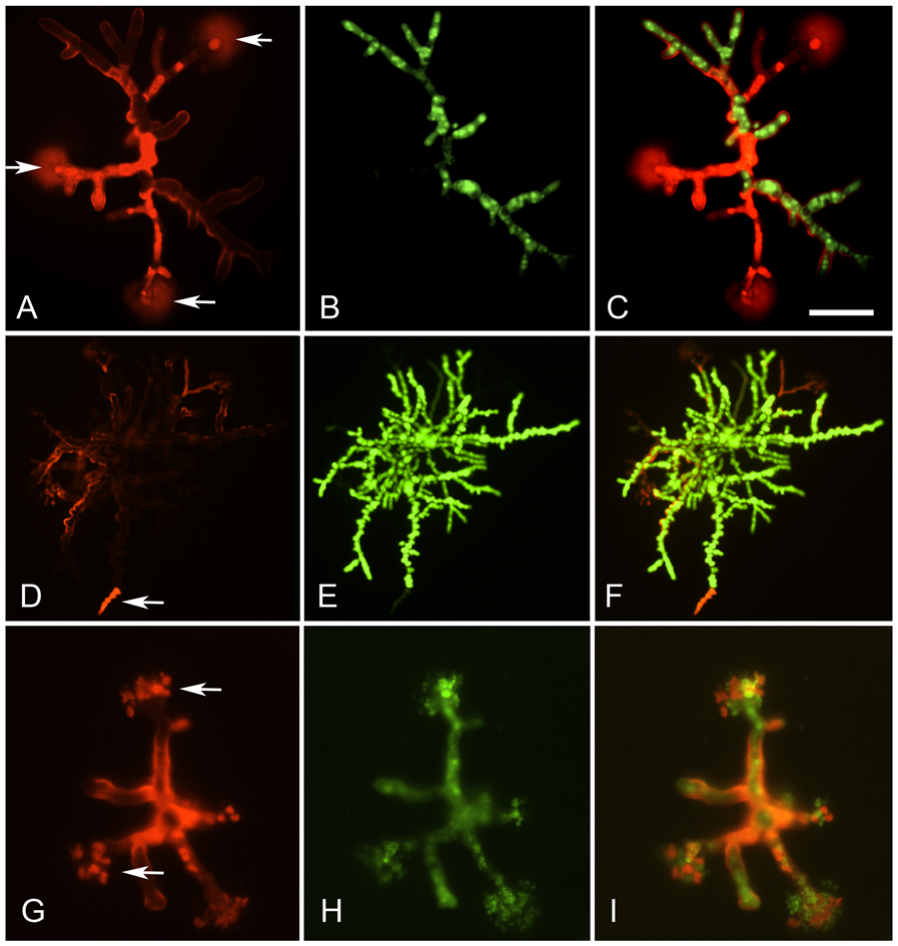
Examples of imaging microcolonies of *Aspergillus spp.* grown on PAO with 0.5 µg/ml caspofungin and dual stained with propidium iodide and Syto9. Culture in all cases was for 14 h at 37°C on Sabouraud medium. Panels A–C; CASP sensitive strain of *A. fumigatus* (JBZ11). A: Staining with PI. B: Staining with Syto9. C: Merged. Arrows in panel A indicate lysed hyphal tips. Panels D–E; as previous panels but imaging an CASP resistant strain of *A. fumigatus* (CWZ1243) showing greater growth and only limited echinocandin mediated damage (e.g. arrow). G–I; as panels A–C, but showing staining of an ANI and CASP sensitive strain of *A. terreus* (CWZ59). Scale bar in panel C indicates 20 µm for panels A–C, 45 µm for panels D–F and 15 µm for G–I.

During culture of sensitive strains with both anidulafungin and caspofungin, lysed hyphal tips were seen (Figure 1B to 1E; Figure 3C and 3D). Lysis could be observed by fluorescence microscopy or by staining microcolonies with Fun-1/calcofluor white (Figure 3) or with Syto9/propidium iodide (Figure 4). Scanning electron microscopy (Figure 1 and Figure 3) confirmed that lysis of hyphal tips was induced by these drugs. Both liquid and vapour fixation methods for SEM preparation gave the same results, with a good correspondence with fluorescence microscopy. In all cases the debris resulting from tip lysis could be observed in an arc pattern radiating outwards from the lysed tip. The material liberated from lysed cells adhered to the PAO, providing a convenient marker for scoring the location of a lytic event. Using fluorescence microscopy (with microcolonies stained with Fun-1 and calcofluor white) the staining pattern with each dye individually indicated that the calcofluor white was responsible for detecting the damage. Therefore, it appears likely that cell wall debris was being stained. With combined Syto9 and propidium iodide, staining was primarily with the latter dye. When Syto9 was used alone it could also facilitate detection of the debris. It is likely, therefore, that nucleic acids liberated from lysed cells were the primary target for propidium iodide staining, which outcompeted the Syto9 in a mixture due to a higher affinity for DNA binding. When caspofungin resistant strains of *A. fumigatus* were tested (CWZ1243 shown, CWZ93 was similar), tip lysis was only rarely observed (Figure 4D–F) at a concentration sufficient to cause widespread lysis in sensitive strains. Taking these results together, we conclude that both anidulafungin and caspofungin caused apical lysis of hyphae in sensitive strains of *A. fumigatus*. A similar level of lysis with both echinocandins was observed for *A. terreus* (e.g. Figure 4G, Figure 4H and Figure 4I).

The concentrations of anidulafungin and caspofungin that triggered the greatest degree of cellular lysis for strain JBZ11 of *A. fumigatus* (0.125 µg/ml and 0.5 µg/ml respectively) were used in Sabouraud agar plates in viable counts. Viability was scored by counting the number of microcolonies that germinated after 14 h. Anidulafungin reduced viability to 86% and caspofungin to 88% relative to the untreated control (mean of 3 determinations). Therefore, the ability of these drugs to prevent microcolony formation was limited. Using higher concentrations (2 µg/ml for both) viable counts were 81% (anidulafungin) and 90% (caspofungin) of the control (n = 3). Therefore, again, at the microcolony level the fungicidal activity of these drugs was marginal. These trends (79 to 89% viability) were similar for other echinocandin sensitive isolates, i.e. strains ATCC204305, JBZ17 and JBZ32 of *A. fumigatus* and strain CWZ59 of *A. terreus*. In contrast, amphotericin B reduced viability <86% when used at the MICs of JBZ11, JBZ13 and JBZ32 (0.05 to 0.1 µg/ml).

#### Quantification of cell lysis by anidulafungin and caspofungin

**Figure 5:**
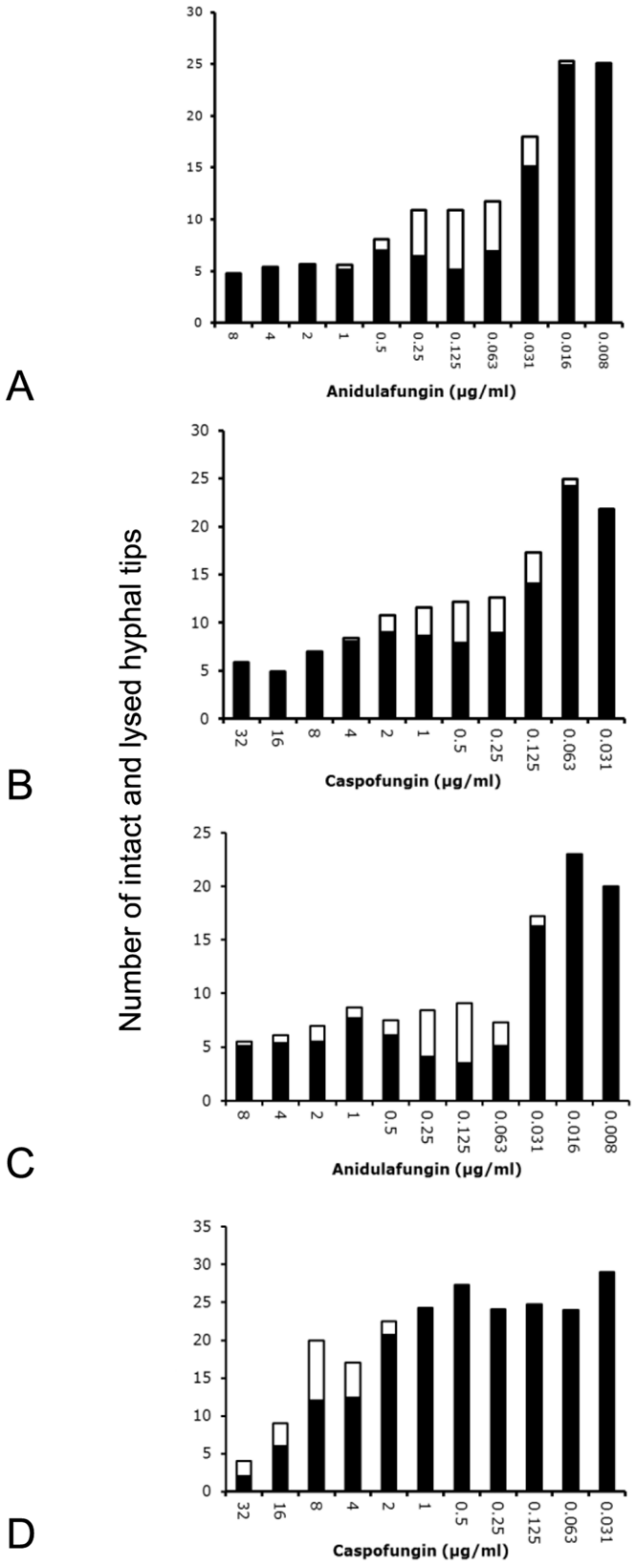
Quantification of the lysis of hyphal tips by echinocandins. Panels A and B: Echinocandin susceptible strain of *A. fumigatus* JBZ11. C and D: Caspofungin resistant, anidulafungin sensitive strain of *A. fumigatus* CWZ1243. Culture was for 14 h at 37°C on Sabouraud medium. A and C: Effect of anidulafungin. Black, average number of intact hyphal tips per microcolony. White, average number of lysed hyphal tips per microcolony. B and D: Effect of caspofungin. Interpretation as panel A.

Cell lysis was scored for a range of concentrations of caspofungin and anidulafungin after 14 h (Figure 5). Lysis was particularly common at intermediate concentrations around the MIC. In contrast, whilst higher concentrations of echinocandins were more effective at limiting growth lysis was seen less often (Figure 5). Anidulafungin appeared somewhat more effective than caspofungin, with >50% cell lysis at the most effective concentrations (0.06 to 0.25 µg/ml). Fungal microcolonies growing on echinocandins appeared heterogeneous in their response. Subpopulations of lysed cells and intact ones coexisted within the same microcolony, ones that were derived, in most cases, from a single conidium. In both cases, a total count of microcolonies after 14 h suggested that neither drug reduced the number of microcolonies, despite having a significant impact on the number of intact hyphal tips within a microcolony. Caspofungin resistance (Figure 5D) increased the concentration of this drug required for optimal tip lysis commensurate with the increased MIC compared to sensitive strains.

**Figure 6:**
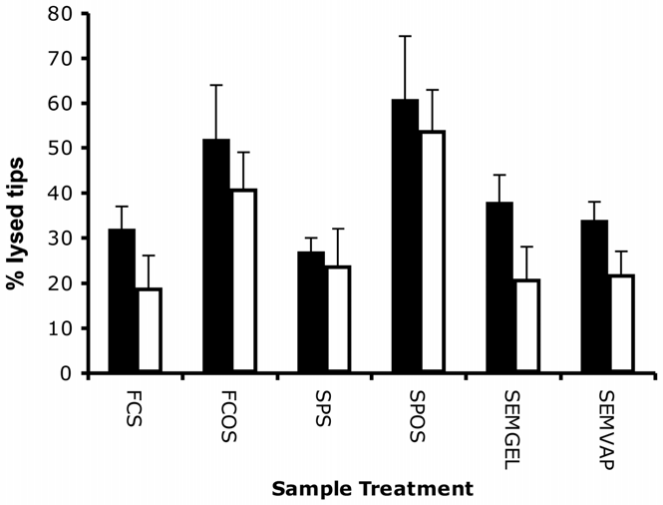
Quantification of the lysis of hyphal tips by echinocandins by sample preparation method. Effect of sample preparation method on percentage lysed hyphal tips. Black bars, 0.125 µg/mlanidulafungin. White bars, 0.5 µg/ml caspofungin. FCS, Fun-1 and calcofluor white staining on Sabouraud medium with imaging by fluorescence microscopy. FCOS, Fun-1 and calcofluor white staining with osmotic shock on water agar and fluorescence microscopy. SPS, Syto9 and propidium iodide staining on Sabouraud medium with assessment by fluorescence microscopy. SPOS, Syto9 and propidium iodide staining with osmotic shock on water agar and fluorescence microscopy. SEMGEL, glutaraldehyde fixation by gel method and imaging by SEM. SEMVAP vapour fixation method and SEM imaging. Error bars indicate S.D. from the mean (n = 3).

A series of control experiments were performed, using different staining or fixing and imaging methods, to check that the observed tip lysis was not affected by sample preparation (Figure 6) or by dye choice. The latter point was considered, in part, as calcofluor white is known to destabilise the cell wall in *A. nidulans* [12]. Fun-1/calcofluor white staining, propidium iodide/Syto9 staining, scanning electron microscopy with vapour or gel fixation all gave similar frequencies of lysis. Therefore it can be concluded that imaging or staining methods did not bias the results. However, staining under conditions of osmotic stress elevated the frequency of lysis to over 60% (Figure 6). This suggests that a subpopulation of cells existed that did not lyse with an echinocandin alone but which were vulnerable when the antifungal agent was combined with an osmotic shock.

#### Correlation between observed lysis and staining with Syto9 or propidium iodide for anidulafungin

Microcolonies of *A. fumigatus* cultured from conidia with echinocandins were analyzed using Syto9/propidium iodide staining, relating the number of lysed and intact hyphal tips per microcolony to the staining pattern. Microcolonies cultured without drugs for 14 h showed no lysis and >98% of hyphal tips (154 out of 157) stained with Syto9. The same analysis was repeated for 0.125 µg/ml anidulafungin, giving maximal lysis. Assessing 198 hyphal tips, 47% (n = 93) showed lysis and 53% (n = 105) were apparently intact. Of the 105 lysed tips imaged, >94% (n = 87) were stained with propidium iodide in preference to Syto9. In contrast, of the 93 apparently intact tips 37% were found to stain with propidium iodide (n = 34) and Syto9 (63%, n = 59). However, some intact tips that stained with propidium iodide were adjacent to lysed tips, and it is likely that many of this subpopulation were part of the same cell or hyphal compartment (Figure 4). When a higher concentration of anidulafungin was used (2 µg/ml) only low rates of lysis were observed (6% of hyphal tips, 10/163). All 10 hyphal tips preferentially stained with propidium iodide. This supports the conclusion obtained from SEM and Fun-1/calcofluor white staining methods; that whilst clearly limited in growth rate *A. fumigatus* growing on high concentrations of anidulafungin on appear less vulnerable to cell death than those growing on an intermediate concentration. In contrast, amphotericin B (at the MICs of each strain, which varied from 0.025 to 0.1 µg/ml) gave <90% propidium iodide staining of cells forthe strains of *A. fumigatus* listed in Table 1. Additionally, little heterogeneity was observed, most microcolonies stained completely with propidium iodide.

#### Recovery of microcolonies of A. fumigatus from the effect of anidulafungin and caspofungin

**Figure 7:**
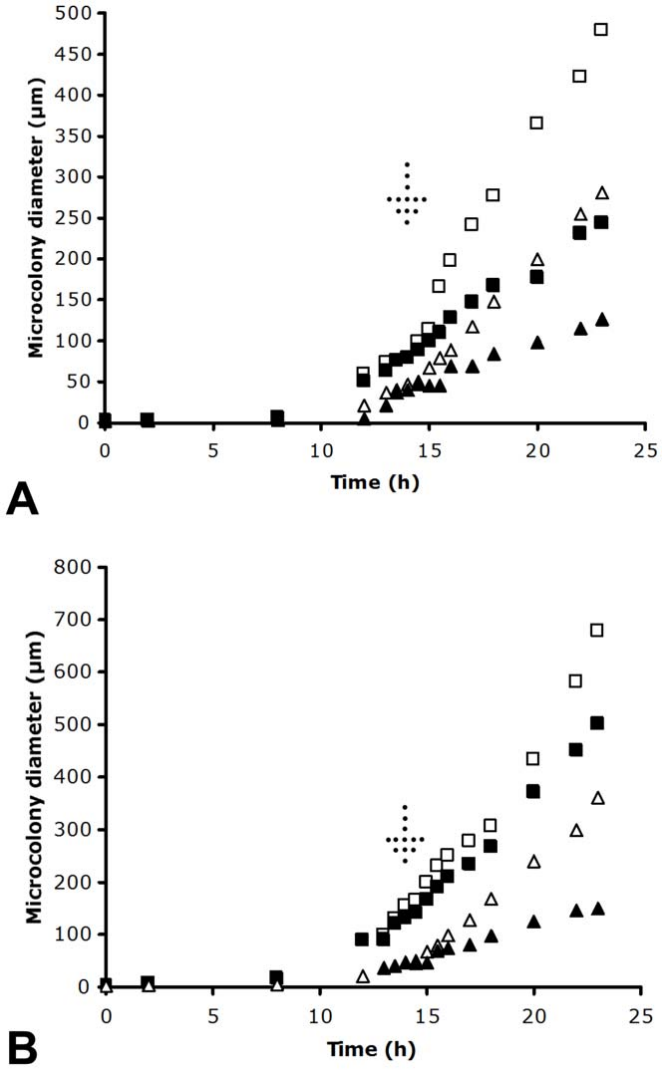
Recovery from echinocandins by sensitive strain JBZ11. A: Recovery from anidulafungin. Open squares, initial growth on 0.125 µg/ml anidulafungin then shift to Sabouraud agar with no drugs at 14 h (indicated by arrow). Open triangles, downshift from 1 µg/ml anidulafungin to none at 14 h. Solid squares, control with growth on 0.125 µg/ml throughout experiment. Solid triangles, continuous growth on 0.125 µg/ml anidulafungin. B: Recovery from caspofungin. Open squares, initial growth on 0.125 µg/ml caspofungin then shift to Sabouraud agar with no drugs at 14 h (indicated by arrow). Open triangles, downshift from 1 µg/ml caspofungin to none at 14 h. Solid squares, control with growth on 0.125 µg/ml throughout the experiment. Solid triangles, continuous growth on 0.125 µg/ml caspofungin.

PAO strips were moved from plates containing echinocandins to those lacking the drugs in order to look at the potential for recovery from echinocandins. Control experiments were first performed by moving uninoculated strips from plates containing echinocandins to drug free plates and then inoculating with *A. fumigatus* conidia. No inhibition of growth was seen, suggesting that the carry-over of drugs within the minimal volume of the PAO pores was not sufficient to influence the results. It was clear from assessment of the average microcolony diameter at different time points that after removal recovery was widespread throughout the population (Figure 7). Microcolonies cultured for 14 h on plates containing 0.125 µg/ml anidulafungin were stained with propidium iodide and Syto9 and compared with those cultured for 17 h on the same concentration or those shifted to zero anidulafungin after 14 h then grown for 3 h. In all cases (>40 observations) lysed hyphal tips stained (>90%) with propidium iodide. Furthermore, there was no evidence for extension of a lysed hyphal tip beyond the debris created by the lysis event. Similar experiments looking at recovery from 0.5 µg/ml caspofungin gave the same result. Therefore, there was no regrowth of lysed cells. Whilst microcolonies containing lysed cells clearly were recovering and growing, this was due to the growth of less damaged cells. This supports other lines of evidence (SEM, fluorescence staining) that echinocandins are fungicidal for specific cells [13]. Because of the overgrowth of lysed hyphae, it was not possible to conclude that lysed cells never recover, but it is likely that this is not a common event.

#### The interaction between Cyclosporin A and echinocandins

**Figure 8:**
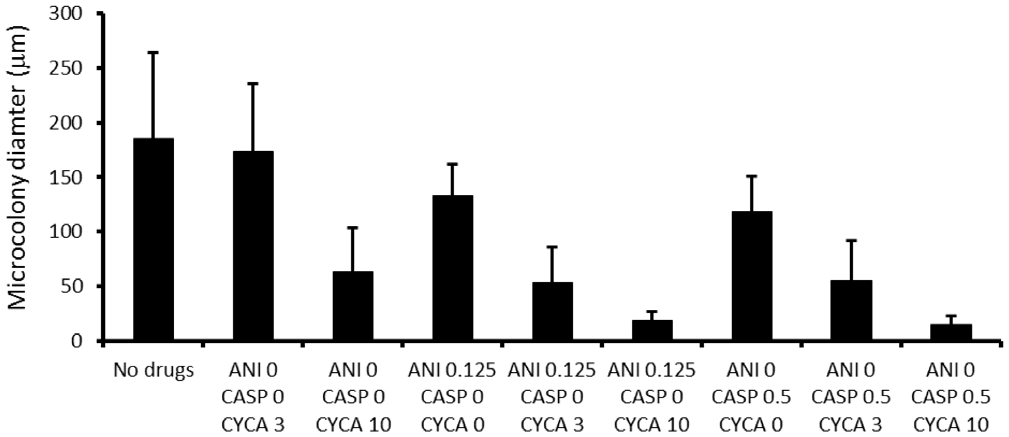
Effect of combining cyclosporine A with anidulofungin or caspofungin on the growth of *A. fumigatus* JBZ11. The average microcolony diameter (+/− S.D.) was determined from >100 microcolonies/data point after 16 h culture on PAO placed on Sabouraud medium containing the drugs. Drug concentrations are given in µg/ml.

The immunosuppressive and calcineurin pathway inhibitor cyclosporine A is known to have fungicidal activity for *A. fumigatus*. Cyclosporine has been shown to combine positively as an antifungal agent with caspofungin against some clinical isolates of *A. fumigatus* [14]. Therefore, this interaction was tested on PAO. After 14 h culture on PAO, cyclosporine A induced rounded cells staining predominantly with Syto 9 when present at concentrations >3 µg/ml. At 3 µg/ml cyclosporine had little effect on cell morphology alone but did enhance the action of echinocandins (Figure 8).

## Discussion

*A. fumigatus* was found to germinate, grow and produce conidia on a PAO support. Conidial germination and hyphal extension and microcolony growth appeared efficient on aluminium oxide, these parameters were similar to growth on agar. The addition of echinocandins allowed the effects of these drugs to be studied at the microscopic level, in microcolonies up to several hundred microns in diameter. As with other culture methods on PAO, imaging by fluorescence microscopy after staining with fluorogenic dyes or processing for SEM was facilitated by the inert support. This has advantages over previous microscopic studies of the effect of caspofungin on cells, with the ready addition and removal of reagents without disturbing the microcolony. Additionally, effective imaging was possible on the planar surface. This permitted relatively high throughput image processing and quantification compared to previous studies, which used liquid culture to investigate caspofungin and micafungin lethality [7], [8]. As far as we are aware, this is the first report of anidulafungin lethality.

Caspofungin is the only echinocandin to have been studied both in liquid culture and on PAO. Broadly, the two studies reveal similar trends, with one exception. Lysis was most apparent on PAO at intermediate concentrations of caspofungin, i.e. those close to MIC values. Higher concentrations of both echinocandins limited microcolony growth to a greater extent but resulted in reduced lysis. This may be due to more rapidly growing cells being more vulnerable to lysis. Paradoxical growth has previously been described for *A. fumigatus* treated with echinocandins, i.e. when very high doses have a lesser effect on growth compared to intermediate dosage. Explanations of paradoxical growth often invoke compensatory mechanisms for limiting cell wall damage, such as the stimulation of chitin synthesis, which reduce drug effects at high concentrations. However, both at the microcolony level, and in E-tests, we did not observe classical paradoxical effects in these studies (in the sense that increasing concentrations echinocandins including those significantly above the MIC decreased the size of microcolonies). In contrast, for liquid culture increasing concentrations of caspofungin above the MIC has been reported to lead to enhanced lysis and cell death [7]. The ability to separate growth inhibition and tip lysis using microcolony imaging should allow paradoxical effects of drugs to be studied effectively in the future.

Apart from the obvious damage, two supporting lines of evidence indicated that cell death was occurring. The first is an excellent correlation between propidium iodide staining and lysis of hyphal tips. So-called “live-dead” staining is commonly used within bacteriology as an indicator of membrane permeability and therefore as an indirect measure of cell death. Whilst this staining method is not commonly used for *Aspergillus*, this dye pairing is sold for yeast viability assays (Funga Light Kit, Invitrogen). Secondly, cell debris from lysed cells adhered to PAO and this lead to a fortuitous and novel marker for the location of a lytic event. This debris was visible when stained with calcofluor white or propidium iodide and could also be imaged by SEM. The distribution of debris was a characteristic arc pattern, suggesting a single, violent lytic event at a single location. Lysed tips did not extend further, i.e. beyond the lysis point, under conditions in which the microcolonies were growing, at least within a few hours of removal of the drug. This suggested that repair and regrowth of lysed hyphal tips did not occur within the time frame of these experiments. Previously, we have also shown that the debris resulting from *Enterobacteriaceae* lysed by trimethoprim could be imaged on PAO [11]. Taken with this work, this supports the idea that more information can be obtained from extremely damaged or highly stressed cells than by many other culture methods.

One way of thinking about the killing efficiency of the echinocandins is that these drugs are not fungicidal at the level that matters, as complete destruction of a microcolony seems very hard to achieve despite the dramatic effect on individual cells. But describing these drugs as fungistatic also seems simplistic for two reasons. The first is that growth of microcolonies never halts completely. More important is the high level of cell death, at least at specific concentrations of echinocandins (in some cases >50% cell lethality). This latter point suggests that looking for treatment regimes, or new echinocandins that are more aggressively fungicidal, may be interesting ways of improving the clinical effectiveness of this group of drugs. For example, the combination of the echinocandin FK463 with chitin synthase inhibitor nikkomycin Z has been reported to be synergistic against *A. fumigatus* when tested *in vitro* [13]. In our study, osmotic shock was shown to increase echinocandin-mediated lysis (Figure 6). We were also able to confirm previous work [14] indicating that the calconeurine pathway inhibitor and immunosuppressive cyclosporine A (Figure 8) enhanced the action of echinocandins. It is possible that dosing methods, complementary drugs or cofactors may be found that improve treatment with existing echinocandins. Currently, some of the clinical effectiveness of the echinocandins against *A. fumigatus in vivo* is attributed to the exposure of cell antigens to the immune system; i.e. indirect effects as well as direct inhibition of fungal growth [6]. It certainly appears possible that greater direct killing by this class of drugs is achievable.

Culture on PAO is relatively simple, with the facility to gain quantitative data on microcolony growth with changes in the environment. Here this method has been used to investigate stress and recovery of a filamentous fungus from a particular class of therapeutic agents, the echinocandins. We suggest that this approach may be more widely usable, for example to investigate fungi difficult to study in other ways because of slow growth and/or limitations of liquid culture methods such as aggregation. It is possible to change the properties of PAO (e.g. porosity, charge, hydrophobicity, texture), which may allow a systematic investigation as to how filamentous fungi are affected by surfaces [15]. Imaging by fluorescence microscopy can detect marginal growth (Figure 1) and recovery from echinocandins (Figure 6). As gradients of drugs can be created beneath PAO supports [16], more complex drug effects can be investigated – for example issues of synergy or antagonism between multiple antibiotics – or growth under extreme stress.

## Methods

### Culture of *A. fumigatus* and *A. terreus* and exposure to drugs on porous aluminium oxide

#### Strains

All strains of *Aspergillus* species used in this study were clinical isolates or reference strains, as detailed in Tables 1 and 2.

#### Culture and Susceptibility Testing

PAO strips were sterilized and handled as previously described [9], [10]. Dispersed preparations of *A. fumigatus* conidia [17] were inoculated onto sterile PAO (placed on the appropriate agar plate) at a density of 10–40 cfu/mm^2^. Culture was at 37°C. PAO strips were transferred between plates using sterile parafilm. Echinocandins were delivered to fungi either at a concentration in the agar plate or by using an E-strip (BioMerieux) aligned with the long axis of a 36 mm×8 mm PAO strip [16]. E-tests were performed directly on Sabouraud agar as recommended by the manufacturer, with 36 h incubation at 37°C. Cyclosporine A was obtained from Sigma Aldrich (NL) and was diluted from a stock solution of 25 mg/ml prepared in methanol. All experiments on growth on PAO, drug effects on PAO and conventional MIC testing were performed in triplicate.

### Recovery of *A. fumigatus* from echinocandins

PAO strips were moved from plates containing echinocandins to those lacking the drugs in order to look at the potential for recovery from echinocandins. Because of the low volume of PAO strips relative to the agar beneath carry-over of drugs to plates lacking echinocandins was minimal (>1000 fold dilution).

### Scanning electron microscopy (SEM)

Fixation of microcolonies for imaging by SEM was by one of two methods: 1) gel fixation was achieved by transferring the strip of PAO to an agar plate containing Sabouraud medium with 1% (v/v) glutaraldehyde for 30 min; 2) vapour fixation was performed by inverting an agar plate above the glutaraldehyde/paraformaldehyde fixative for 2 h [18]. In both cases, treatment with osmium tetroxide, ethanol dehydration, critical point drying, sputtering with tungsten and imaging by an FEI Magellan electron microscope were as previously described [11].

### Staining and fluorescence microscopy

Staining of *A. fumigatus* and *A. terreus* with pairs of dyes (Fun-1/calcofluor white or syto9/propidium iodide) was performed by transferring the PAO strip to a microscope slide covered in a thin layer of low melting point agar containing the dye. Dye concentrations and staining times were as previously described [9]. The low melting point agar was formulated with water to deliver an osmotic shock or with Sabouraud medium to stain under the same nutrient conditions as growth.

### Quantification and data analysis

#### Image processing

TIFF format images were processing using ImageJ software (Rasband WS, 1997–2011 http://rsbweb.nih.gov/ij/). Images were inspected individually and only images with well separated microcolonies processed. Multiple images were then assembled into stacks for batch processing using an ImageJ macro that performed the operations of: [A] application of a median filter (pixel radius 3), [B] thresholding to black microcolonies on a white background and [C] using the “analyze particle” function to determine the dimensions (average diameter, area) of each microcolony within the field of view. Objects only partially within a field of view were excluded from this analysis. Datasets were exported into Microsoft Excel for further calculations.

#### Calculations of tip lysis and Syto9 vs propidium iodide staining

The frequency of lysed and unlysed tips was calculated from at least 50 microcolonies per condition. Hyphal tips <4 µm in length were not included in this analysis. Cells including hyphal tips were scored as Syto9 if the staining pattern was more intense than the competitor dye propidium iodide. Cells for which the converse was true were scored as propidium iodide staining.

#### Statistics and calculation of variance

Statistical operations (ANOVA, Student's t-test, determinations of normality) used the Vassar Statistics web server (Lowry RS, 1998–2011 http://faculty.vassar.edu/lowry/VassarStats.html). Microcolony heterogeneity was assessed using log_10_ transformations of variance in microcolony area and diameter [19].
